# Synthesis and crystal structure studies of 5-(tri­fluoro­meth­yl)-1,3,4-thia­diazol-2(3*H*)-one at 180 K

**DOI:** 10.1107/S2056989023004267

**Published:** 2023-05-19

**Authors:** Doreswamy Geetha, Thaluru M. Mohan Kumar, Haleyur G. Anil Kumar, Mellekatte T. Shreenivas, Yeriyur B. Basavaraju, Hemmige S. Yathirajan, Sean Parkin

**Affiliations:** a Department of Studies in Chemistry, University of Mysore, Manasagangotri, Mysuru-570 006, India; b Department of Chemistry, Amrita School of Engineering, Amrita Vishwa Vidyapeetham, Bengaluru-560 035, India; c Department of Science and Humanities, PES University, BSK III Stage, Bengaluru-560 085, India; d Honeychem Pharma Research Pvt. Ltd., Peenya Industrial Area, Bengaluru-560 058, India; e Department of Chemistry, University of Kentucky, Lexington, KY, 40506-0055, USA; Katholieke Universiteit Leuven, Belgium

**Keywords:** 1,3,4-thia­diazole, heterocycle, high *Z*′, *Z*′ = 6, hydrogen bonding, disorder, crystal structure

## Abstract

The synthesis and crystal structure of C_3_HF_3_N_2_OS, systematic name 5-(tri­fluoro­meth­yl)-1,3,4-thia­diazol-2(3*H*)-one (5-TMD-2-one), a compound containing the pharmacologically important heterocycle 1,3,4-thia­diazole, is presented. The asymmetric unit comprises six independent mol­ecules (*Z*′ = 6), all of which are planar. The r.m.s. deviations from each mean plane range from 0.0063 to 0.0381 Å, not including the CF_3_ fluorine atoms. Within the crystal, two of the mol­ecules form hydrogen-bonded dimers that in turn combine with inversion-related copies to form tetra­meric constructs. Similar tetra­mers, but lacking inversion symmetry, are formed by the remaining four mol­ecules. The tetra­mers are linked into tape-like motifs by S⋯O and O⋯O close contacts. The environments of each symmetry-independent mol­ecule were compared *via* a Hirshfeld surface analysis. The most abundant atom–atom contacts are between fluorine atoms, while the strongest result from N—H⋯O hydrogen bonds.

## Abstract 2

The synthesis and crystal structure of 5-(tri­fluoro­meth­yl)-1,3,4-thia­diazol-2(3*H*)-one, a heterocycle of importance as a pharmaceutical building block, are presented.

## 1. Chemical context

The 1,3,4-thia­diazole ring is a pharmacologically important heterocycle found in compounds covering a broad spectrum of bioactivity (Moussa *et al.*, 2023 ▸). Recent reviews have highlighted the beneficial properties of 1,3,4-thia­diazole derivatives, including microbiological activity (Barbosa & de Aguiar, 2019 ▸) and their potential use as scaffolds for drug design and development (Han *et al.*, 2021 ▸). A series of 2,5-disubstituted 1,3,4-thia­diazole derivatives were synthesized and investigated for anti­tuberculosis structure–activity relationships by Oruç *et al.* (2004 ▸). The structures and thermal behaviour of substituted 1,3,4-thia­diazole organic crystals have been investigated by Shen *et al.* (2005 ▸). Reviews of progress covering the biological activities of 1,3,4-thia­diazole and its derivatives were reported by Jain *et al.* (2013 ▸) and by Anthwal *et al.* (2022 ▸). Their use as scaffolds for promising anti­microbial agents (Serban *et al.*, 2018 ▸) and anti-cancer agents (Çevik *et al.*, 2020 ▸) have also been published. The inter­play of inter- and intra­molecular inter­actions in the crystal structures of 1,3,4-thia­diazole resorcinol derivatives was reported by Hoser *et al.* (2018 ▸). A series of four biologically active 2-benzamido-5-(4-fluoro-3-phen­oxy­phen­yl)-1,3,4-thia­diazo­les derivatives were synthesized by Panini *et al.* (2013 ▸) and their crystal structures studied to evaluate the effects of systematic variations in the functional group attached at the *para* position of the benzamido ring. Lastly, the crystal structures of three 6-aryl-2-(4-chloro­benz­yl)-5-[(1*H*-indol-3-yl)meth­yl]imidazo[2,1-*b*][1,3,4]thia­diazo­les were reported by Shamanth *et al.* (2020 ▸).

Overall, the 1,3,4-thia­diazole heterocycle provides the basis of a promising area of research in medicinal chemistry and drug discovery, with a wide range of potential applications. The reported findings provide insights into the mol­ecular properties and biological activities of 1,3,4-thia­diazole derivatives, contributing to the development of novel therapeutic agents. With the importance of 1,3,4-thia­diazo­les in drug discovery research in mind, this paper reports the synthesis and crystal structure of 5-(tri­fluoro­meth­yl)-1,3,4-thia­diazol-2(3*H*)-one, C_3_HF_3_N_2_OS (5-TMD-2-one). 
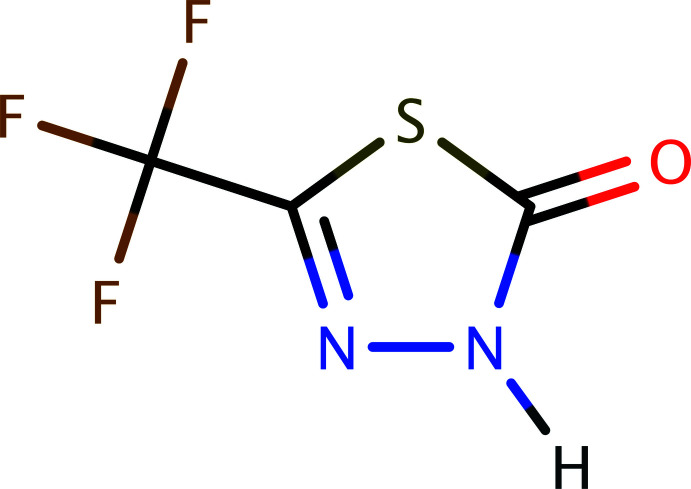


## 2. Structural commentary

**Figure 1:**
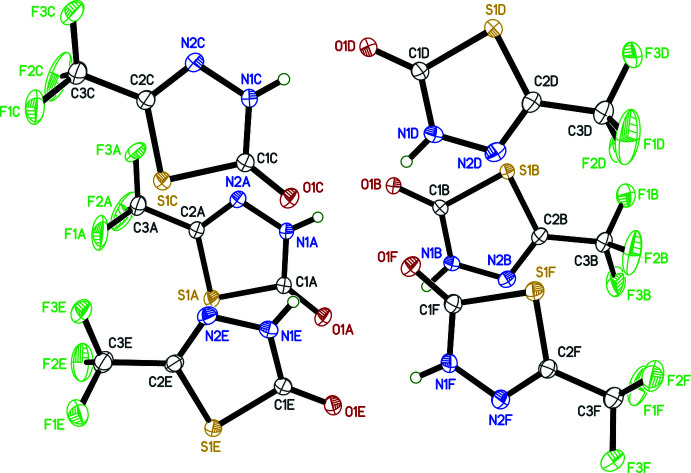
An ellipsoid plot (30% probability) of the asymmetric unit of 5-TMD-2-one. The CF_3_ groups on all six independent mol­ecules are disordered over two orientations, but only the major components are shown.

The mol­ecular structure of 5-TMD-2-one consists of a 1,3,4-thia­diazone ring, essentially a flat penta­gonal heterocycle with two adjacent nitro­gen atoms, each flanked by carbon atoms, with a sulfur atom completing the ring. The simplicity of the mol­ecular structure notwithstanding, the crystal structure of 5-TMD-2-one is far more complex, as the asymmetric unit contains six mol­ecules (*Z*′ = 6; designated *A*–*F* in Fig. 1 ▸). In each mol­ecule, one of the nitro­gen atoms (N1) carries a hydrogen atom and is single bonded to C1, while N2 is double bonded to C2. Atom C1 forms a carbonyl group with O1, and C2 carries a tri­fluoro­methyl substituent. Deviations (r.m.s.) from planarity range from 0.0063 Å in mol­ecule *B* to 0.0381 Å in mol­ecule *D*, with the largest deviation for any atom (aside from fluorine), being 0.065 (8) Å for C3*D*, the tri­fluoro­methyl carbon of mol­ecule *D*. The only inter­nal degree of freedom is the torsion of the tri­fluoro­methyl group, which is disordered in all six symmetry-independent mol­ecules in the structure. Indeed, the CF_3_ orientations and the refined occupancies of the disorder components, which range from 0.500 (5):0.500 (5) for mol­ecule *D* to 0.908 (2):0.092 (2) for mol­ecule *F*, are the only significant differences between the six mol­ecules.

The crystals were observed to shatter when cooled to 90 K, but remained intact and gave sharp diffraction at 180 K. This observation prompted us to investigate whether warming the crystals might lead to a simpler crystal structure, *i.e.*, with fewer mol­ecules in the asymmetric unit. A crystal mounted at room temperature, however, indexed to give essentially the same unit cell and structure (again with *Z*′ = 6) as at 180 K.

## 3. Supra­molecular features

**Figure 2:**
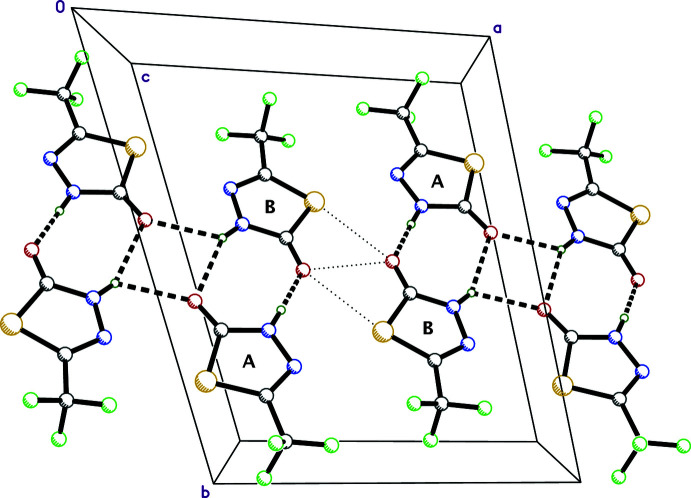
A partial packing plot of 5-TMD-2-one viewed down the *c*-axis for the *A* and *B* mol­ecules, showing N—H⋯O hydrogen bonds (dashed lines) and inter­molecular contacts (S⋯O and O⋯O, dotted lines), forming a tape-like motif parallel to (011) that extends along the *a*-axis direction. The hydrogen bonding and inter­molecular contacts for mol­ecules *C*, *D*, *E* and *F* are similar, but lack crystallographically imposed inversion symmetry.

**Figure 3:**
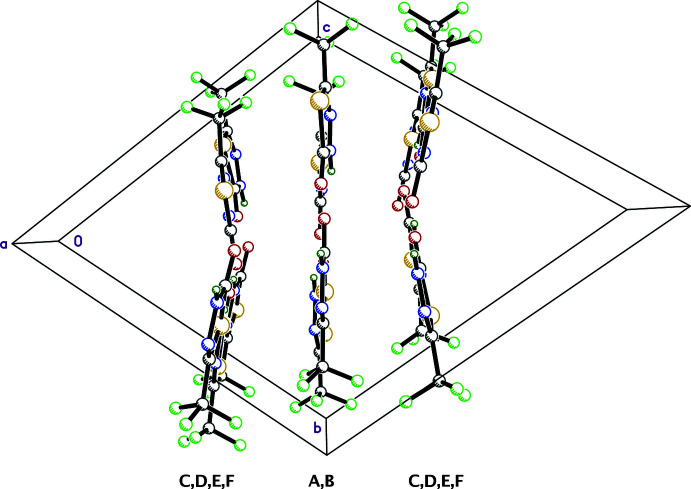
A partial packing plot of 5-TMD-2-one viewed down the *a*-axis, showing the main difference between the *A*/*B* tape motif and those formed by mol­ecules *C*, *D*, *E* and *F*, which have a shallow V-shaped cross section.

The main supra­molecular constructs in crystals of 5-TMD-2-one are hydrogen-bonded tetra­mers. There are, however, slight differences for tetra­mers formed by mol­ecules *A* and *B* (with inversion-related copies) and by mol­ecules *C*, *D*, *E* and *F*. Within the chosen asymmetric unit, mol­ecules *A* and *B* are joined by one short N1*A*—H1*A*⋯O1*B* [*d_D–A_* = 2.726 (2) Å] and one longer N1*B*—H1*B*⋯O1*A* [*d_D–A_* = 3.328 (2) Å] hydrogen bond, leading to 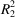 (8) dimer motifs. Pairs of these dimers are connected to inversion-related copies by N1*B*—H1*B*⋯O1*A* ^i^ [*d_D–A_* = 2.955 (2) Å; symmetry code: (i) 2 − *x*, 1 − *y*, 1 − *z*] hydrogen bonds, producing 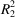 (4) motifs in which the hydrogen atoms act as bifurcated donors (Fig. 2 ▸), thereby generating tetra­mers. Adjacent tetra­mers of *A* and *B* mol­ecules are in close contact [*via* S1*B*⋯O1*B* ^ii^ = 2.9743 (14) Å and O1*B*⋯O1*B* ^ii^ = 2.996 (3) Å; symmetry code: (ii) 1 − *x*, 1 − *y*, 1 − *z*] contacts, forming tape-like structures parallel to (011) that extend along the [100] direction. For mol­ecules *C*, *D*, *E* and *F*, the individual motifs are similar (see Table 1 ▸), but lack the constraints of inversion symmetry, leading to tapes with a slightly V-shaped cross section, as shown in Fig. 3 ▸. Owing to the complexity, however, the overall packing is best viewed using a mol­ecular graphics program such as *Mercury* (Macrae *et al.*, 2020 ▸). Hydrogen bonding and close-contact distances are given in Table 1 ▸.

**Figure 4:**
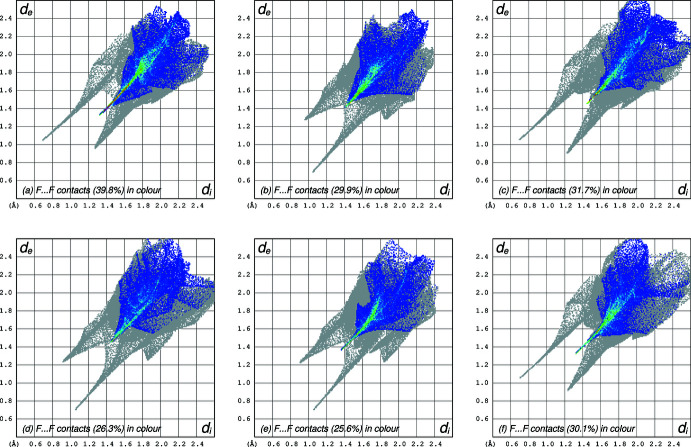
Hirshfeld surface two-dimensional fingerprint plots for each of the six independent mol­ecules *A*–*F* [depicted in panels (*a*)–(*f*)] of 5-TMD-2-one. The F⋯F contacts, highlighted in blue and green have the greatest coverage. The N—H⋯O hydrogen bonds are apparent as grey spikes extending to the lower left in each panel. The longer, sharper spikes correspond to the shorter, stronger inter­actions in each case.

Atom–atom contact two-dimensional fingerprint plots calculated using *CrystalExplorer* (Spackman *et al.*, 2021 ▸) for each of the six independent mol­ecules show that their environments are similar (Fig. 4 ▸ *a*–*f*). The most abundant contacts in each case are F⋯F (shown in blue and green), ranging from 39.8% in mol­ecule *A* (Fig. 4 ▸ *a*) to 25.6% in mol­ecule *E* (Fig. 4 ▸ *e*).

## 4. Database survey

A search of the Cambridge Structural Database (CSD, v5.43 with updates to November 2022; Groom *et al.*, 2016 ▸) for ‘thia­diazole’ returned 2068 hits, while ‘1,3,4-thia­diazole’ gave 745 hits. A subsequent search using just the 1,3,4-thia­diazole ring fragment with ‘any substituent’ specified at the equivalent of C1, C2, and N1 produced 682 hits. This fragment, but with hydrogen attached to N1 gave 114 hits. A search with tri­fluoro­methyl added at C2 gave no hits, while a search with ‘any oxygen-bound’ substituent on C1 returned only four hits. These are GAQVIF (Zhang *et al.*, 2012 ▸), which is 5-meth­oxy-1,3,4-thia­diazol-2(3*H*)-one, LAPSAY (Kang *et al.*, 2012*a* ▸), which is a DMSO solvate of 5,5′-[1,4-phenyl­enebis(methyl­enesulfanedi­yl)]bis­[1,3,4-thia­diazol-2(3*H*)-one], and triclinic (YAXWAX: Kang *et al.*, 2012*b* ▸) and monoclinic (YAXWAX01: Kim & Kang, 2014 ▸) polymorphs of 5-amino-1,3,4-thia­diazol-2(3*H*)-one.

A few other crystal structures of compounds related to 5-TMD-2-one include MAZZIX and NIYDOO01 (Boechat *et al.*, 2006 ▸), 1,3,4-thia­diazo­lium-2-thiol­ate (Hu *et al.*, 2006 ▸) and 3-(mercaptometh­yl)-1,3,4-thia­diazol-2(3*H*)-one (HORZAQ; Hartung *et al.*, 2009 ▸).

Although crystal structures with *Z*′ > 1 are not uncommon, their scarcity increases with *Z*′. In a detailed survey of structures with high *Z*′, Brock (2016 ▸) estimated that only about 12% of structures in the Cambridge Structural Database at the time (CSD; Groom *et al.*, 2016 ▸) had *Z*′ > 1, and < 0.1% had *Z*′ > 4. Without any attempt to filter duplicates or pathological cases, in the current version of the CSD (v5.43, *vide supra*) there are 655 entries with *Z*′ = 6 out of over 1.2 million (∼0.05%), so by this criterion alone, the structure of 5-TMD-2-one is unusual, though not unprecedented.

## 5. Synthesis, crystallization and spectroscopic details

*Synthesis of 2-amino-5-tri­fluoro­methyl-1,3,4-thia­diazole*

To a clean and dry 1 L round-bottom flask, 14.5 g of thio­semicarbazide suspended in 500 ml of 1,4 dioxane was added, with stirring. 12.0 ml of CF_3_COOH and 15.0 mL of POCl_3_ were slowly added over about 30 min. The reaction was maintained for 3 h, during which time, a large amount of HCl gas was produced. After completion of HCl gas liberation, the reaction mixture was poured into 100 mL of cold water with stirring and the pH adjusted to 9 with 50% NaOH solution, to give a solid precipitate. The product, 2-amino-5-tri­fluoro­methyl-1,3,4-thia­diazole, was filtered, washed with cold water and dried at 363 K (20.6 g).

*Synthesis of 5-TMD-2-one*

In a 250 ml round-bottomed flask, 20.6 g of 2-amino-5-tri­fluoro­methyl-1,3,4-thia­diazole was suspended with 150 ml conc. hydro­chloric acid, with stirring. The reaction mixture was cooled to between 263 and 268 K. Then, 350 mL of aqueous NaNO_2_ were added slowly (21.2 g, 0.307 mol, 4 eq.) while maintaining the temperature at 263–268 K with continued stirring. After 2 h, 100 ml of H_2_O were added with warming up to 333–353 K and stirred for a further 3 h. The reaction mixture was then cooled to room temperature, 150 mL of CH_2_Cl_2_ were added, the organic layer separated and a further 150 ml CH_2_Cl_2_ were added. The combined organic layers were washed with water twice and dried with sodium sulfate, then finally distilled completely. The crude product was purified by chromatography over SiO_2_ (hexa­ne:EtOAc, 9:1). The resulting product, pure 5-TMD-2-one (12.5 g) was recrystallized from hexane. MS *m*/*z*: 169.12 (*M* − H)+.

**Figure 5:**
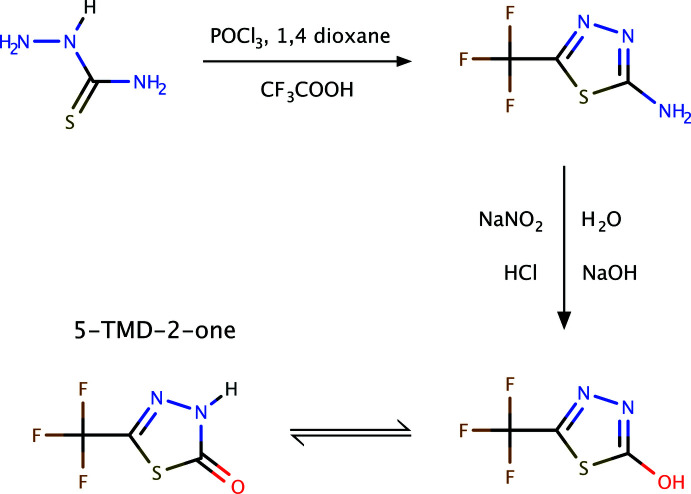
A general scheme for the synthesis of 5-TMD-2-one.

A generalized reaction scheme is presented in Fig. 5 ▸.

## 6. Refinement

Crystal data, data collection and structure refinement details are summarized in Table 2 ▸. All hydrogen atoms were found in difference-Fourier maps. Their coordinates were refined freely with *U*_iso_ parameters set to 1.2*U*_eq_ of their attached nitro­gen atom. To ensure satisfactory refinement of the disordered CF_3_ groups, a combination of constraints (EADP in *SHELXL*) and restraints (*SHELXL* commands SAME, SADI, SIMU, and RIGU) were employed.

## Supplementary Material

Crystal structure: contains datablock(s) I, global. DOI: 10.1107/S2056989023004267/vm2283sup1.cif

Structure factors: contains datablock(s) I. DOI: 10.1107/S2056989023004267/vm2283Isup2.hkl

Supporting information file. DOI: 10.1107/S2056989023004267/vm2283Isup3.cml

CCDC reference: 2263179

Additional supporting information:  crystallographic information; 3D view; checkCIF report

## Figures and Tables

**Table 1:** Hydrogen bonds and other inter­molecular contacts (Å, °) for 5-TMD-2-one

*D*—H⋯*A*	*D*—H	H⋯*A*	*D*⋯*A*	*D*—H⋯*A*
N1*A*—H1*A*—O1*B*	0.819 (16)	1.918 (16)	2.726 (2)	169 (2)
N1*B*—H1*B*—O1*A*	0.826 (15)	2.574 (17)	3.328 (2)	152.3 (19)
N1*B*—H1*B*—O1*A* ^i^	0.826 (15)	2.347 (19)	2.955 (2)	131.0 (18)
N1*C*—H1*C*—O1*D*	0.829 (15)	1.927 (16)	2.7485 (19)	171 (2)
N1*D*—H1*D*—O1*C*	0.821 (15)	2.605 (17)	3.342 (2)	150 (2)
N1*D*—H1*D*—O1*F*	0.821 (15)	2.280 (19)	2.908 (2)	134 (2)
N1*E*—H1*E*—O1*C*	0.814 (15)	2.262 (19)	2.912 (2)	137 (2)
N1*E*—H1*E*—O1*F*	0.814 (15)	2.643 (18)	3.359 (2)	148 (2)
N1*F*—H1*F*—O1*E*	0.820 (16)	1.952 (16)	2.764 (2)	171 (2)
S1*B*⋯O1*B* ^ii^			2.9743 (14)	
O1*B*⋯O1*B* ^ii^			2.996 (3)	
S1*D*⋯O1*E* ^iii^			3.0279 (14)	
O1*D*⋯O1*E* ^iii^			3.0686 (18)	
O1*D*⋯S1*E* ^iii^			3.0093 (14)	

Symmetry codes: (i) −*x* + 2, −*y* + 1, −*z* + 1; (ii) −*x* + 1, −*y* + 1, −*z* + 1; (iii) *x* − 1, *y*, *z*.

**Table 2:** Experimental details

Crystal data	
Chemical formula	C_3_HF_3_N_2_OS
*M*_r_	170.12
Crystal system, space group	Triclinic, *P* 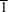
Temperature (K)	180
*a*, *b*, *c* (Å)	10.8996 (7), 13.9700 (8), 14.1351 (9)
α, β, γ (°)	63.253 (2), 71.160 (2), 67.954 (2)
*V* (Å^3^)	1750.38 (19)
*Z*	12
Radiation type	Mo *K*α
μ (mm^−1^)	0.54
Crystal size (mm)	0.32 × 0.29 × 0.28

Data collection	
Diffractometer	Bruker D8 Venture dual source
Absorption correction	Multi-scan (*SADABS*; Krause *et al.*, 2015 ▸)
*T*_min_, *T*_max_	0.922, 0.971
No. of measured, independent and observed [*I* > 2σ(*I*)] reflections	52212, 8021, 6646
*R*_int_	0.045
(sin θ/λ)_max_ (Å^−1^)	0.650

Refinement	
*R*[*F* ^2^ > 2σ(*F* ^2^)], *wR*(*F* ^2^), *S*	0.037, 0.090, 1.02
No. of reflections	8021
No. of parameters	728
No. of restraints	273
H-atom treatment	Only H-atom coordinates refined
Δρ_max_, Δρ_min_ (e Å^−3^)	0.41, −0.31

Computer programs: *APEX3* (Bruker, 2016 ▸), *SHELXT* (Sheldrick, 2015*a* ▸), *SHELXL2019/2* (Sheldrick, 2015*b* ▸), *XP* in *SHELXTL* (Sheldrick, 2008 ▸), *SHELX* (Sheldrick, 2008 ▸) and *publCIF* (Westrip, 2010 ▸).

## supplementary crystallographic information

### Crystal data

**Table:**

C_3_HF_3_N_2_OS	*Z* = 12
*M**_r_* = 170.12	*F*(000) = 1008
Triclinic, *P*1	*D*_x_ = 1.937 Mg m^−^^3^
*a* = 10.8996 (7) Å	Mo *K*α radiation, λ = 0.71073 Å
*b* = 13.9700 (8) Å	Cell parameters from 9954 reflections
*c* = 14.1351 (9) Å	θ = 2.4–33.1°
α = 63.253 (2)°	µ = 0.54 mm^−^^1^
β = 71.160 (2)°	*T* = 180 K
γ = 67.954 (2)°	Block, colourless
*V* = 1750.38 (19) Å^3^	0.32 × 0.29 × 0.28 mm

### Data collection

**Table:**

Bruker D8 Venture dual source diffractometer	8021 independent reflections
Radiation source: microsource	6646 reflections with *I* > 2σ(*I*)
Detector resolution: 7.41 pixels mm^-1^	*R*_int_ = 0.045
φ and ω scans	θ_max_ = 27.5°, θ_min_ = 2.1°
Absorption correction: multi-scan (*SADABS*; Krause *et al.*, 2015)	*h* = −14→14
*T*_min_ = 0.922, *T*_max_ = 0.971	*k* = −17→18
52212 measured reflections	*l* = −18→18

### Refinement

**Table:**

Refinement on *F*^2^	Secondary atom site location: difference Fourier map
Least-squares matrix: full	Hydrogen site location: difference Fourier map
*R*[*F*^2^ > 2σ(*F*^2^)] = 0.037	Only H-atom coordinates refined
*wR*(*F*^2^) = 0.090	*w* = 1/[σ^2^(*F*_o_^2^) + (0.0319*P*)^2^ + 1.258*P*] where *P* = (*F*_o_^2^ + 2*F*_c_^2^)/3
*S* = 1.02	(Δ/σ)_max_ = 0.001
8021 reflections	Δρ_max_ = 0.41 e Å^−^^3^
728 parameters	Δρ_min_ = −0.31 e Å^−^^3^
273 restraints	Extinction correction: *SHELXL2019/2* (Sheldrick, 2015b), Fc^*^=kFc[1+0.001xFc^2^λ^3^/sin(2θ)]^-1/4^
Primary atom site location: structure-invariant direct methods	Extinction coefficient: 0.0078 (13)

### Special details

**Table:**

Experimental. The crystal was mounted using polyisobutene oil on the tip of a fine glass fibre, which was fastened in a copper mounting pin with electrical solder. It was placed directly into the cold gas stream of a liquid-nitrogen based cryostat (Hope, 1994; Parkin & Hope, 1998). The crystals appeared to undergo a destructive phase transition when cooled to 90K. Visual inspection of crystal integrity and diffraction quality vs temperature established a safe temperature for data collection of -93° C.
Geometry. All esds (except the esd in the dihedral angle between two l.s. planes) are estimated using the full covariance matrix. The cell esds are taken into account individually in the estimation of esds in distances, angles and torsion angles; correlations between esds in cell parameters are only used when they are defined by crystal symmetry. An approximate (isotropic) treatment of cell esds is used for estimating esds involving l.s. planes.
Refinement. Refinement progress was checked using *Platon* (Spek, 2020) and by an *R*-tensor (Parkin, 2000). The final model was further checked with the IUCr utility *checkCIF*.

### Fractional atomic coordinates and isotropic or equivalent isotropic displacement parameters (Å^2^)

**Table:**

	*x*	*y*	*z*	*U*_iso_*/*U*_eq_	Occ. (<1)
S1A	0.96653 (5)	0.22146 (4)	0.77875 (4)	0.03426 (12)	
O1A	0.95363 (14)	0.40427 (12)	0.59471 (11)	0.0408 (3)	
N1A	0.76657 (16)	0.34097 (14)	0.68704 (13)	0.0350 (4)	
H1A	0.720 (2)	0.3890 (16)	0.6429 (16)	0.042*	
N2A	0.71853 (16)	0.25921 (14)	0.77307 (13)	0.0365 (4)	
C1A	0.89728 (18)	0.33853 (15)	0.66966 (14)	0.0286 (4)	
C2A	0.81231 (18)	0.19210 (15)	0.82619 (14)	0.0294 (4)	
C3A	0.7884 (12)	0.0917 (7)	0.9244 (7)	0.0411 (6)	0.731 (9)
F1A	0.8079 (7)	0.0963 (5)	1.0078 (2)	0.1020 (18)	0.731 (9)
F2A	0.8728 (5)	0.0021 (2)	0.9115 (4)	0.1037 (19)	0.731 (9)
F3A	0.6673 (3)	0.0848 (4)	0.9448 (4)	0.0895 (16)	0.731 (9)
C3A'	0.792 (3)	0.0889 (17)	0.9217 (17)	0.0411 (6)	0.269 (9)
F1A'	0.7891 (17)	0.0107 (7)	0.8980 (6)	0.081 (4)	0.269 (9)
F2A'	0.6812 (11)	0.1049 (7)	0.9895 (8)	0.084 (4)	0.269 (9)
F3A'	0.8842 (9)	0.0400 (8)	0.9797 (8)	0.068 (3)	0.269 (9)
S1B	0.55246 (4)	0.65799 (4)	0.34668 (4)	0.03139 (12)	
O1B	0.63289 (14)	0.48600 (12)	0.52132 (12)	0.0451 (4)	
N1B	0.77624 (15)	0.59413 (13)	0.40477 (12)	0.0291 (3)	
H1B	0.8414 (18)	0.5583 (16)	0.4356 (16)	0.035*	
N2B	0.78780 (15)	0.68295 (13)	0.31098 (12)	0.0313 (3)	
C1B	0.65984 (18)	0.56361 (15)	0.44064 (15)	0.0300 (4)	
C2B	0.67908 (18)	0.72306 (15)	0.27328 (14)	0.0291 (4)	
C3B	0.6649 (7)	0.8230 (4)	0.1690 (4)	0.0376 (6)	0.802 (7)
F1B	0.5873 (4)	0.8191 (3)	0.1169 (2)	0.0688 (9)	0.802 (7)
F2B	0.6098 (5)	0.91515 (18)	0.1856 (2)	0.0919 (15)	0.802 (7)
F3B	0.7804 (2)	0.8280 (3)	0.1034 (2)	0.0872 (14)	0.802 (7)
C3B'	0.657 (3)	0.8255 (15)	0.1755 (15)	0.0376 (6)	0.198 (7)
F1B'	0.7410 (13)	0.8816 (9)	0.152 (1)	0.069 (4)	0.198 (7)
F2B'	0.6675 (19)	0.8020 (9)	0.0960 (8)	0.076 (4)	0.198 (7)
F3B'	0.5395 (10)	0.8927 (9)	0.1870 (9)	0.073 (3)	0.198 (7)
S1C	0.51557 (5)	0.34764 (4)	1.01026 (4)	0.03561 (13)	
O1C	0.52164 (14)	0.50625 (12)	0.80892 (11)	0.0396 (3)	
N1C	0.33717 (16)	0.43878 (13)	0.89515 (13)	0.0323 (4)	
H1C	0.295 (2)	0.4746 (17)	0.8448 (15)	0.039*	
N2C	0.28288 (16)	0.36498 (13)	0.98623 (13)	0.0338 (4)	
C1C	0.46203 (18)	0.44504 (15)	0.88628 (14)	0.0285 (4)	
C2C	0.36572 (18)	0.31244 (15)	1.05150 (14)	0.0297 (4)	
C3C	0.3342 (8)	0.2217 (5)	1.1581 (4)	0.0396 (6)	0.789 (5)
F1C	0.3650 (4)	0.2303 (2)	1.23606 (15)	0.0761 (10)	0.789 (5)
F2C	0.4036 (4)	0.12531 (17)	1.1556 (2)	0.0997 (16)	0.789 (5)
F3C	0.2062 (2)	0.2288 (3)	1.1867 (2)	0.0937 (14)	0.789 (5)
C3C'	0.335 (3)	0.2182 (18)	1.1521 (14)	0.0396 (6)	0.211 (5)
F1C'	0.2788 (13)	0.1565 (8)	1.1434 (6)	0.069 (3)	0.211 (5)
F2C'	0.2543 (14)	0.2491 (6)	1.2276 (7)	0.079 (4)	0.211 (5)
F3C'	0.4371 (9)	0.1531 (10)	1.1961 (9)	0.084 (4)	0.211 (5)
S1D	0.12479 (5)	0.74725 (4)	0.55582 (4)	0.03674 (13)	
O1D	0.22315 (13)	0.56130 (11)	0.71384 (11)	0.0359 (3)	
N1D	0.34765 (15)	0.68830 (13)	0.61291 (13)	0.0316 (3)	
H1D	0.4136 (18)	0.6541 (17)	0.6423 (17)	0.038*	
N2D	0.34835 (16)	0.78693 (14)	0.52875 (13)	0.0367 (4)	
C1D	0.23960 (18)	0.64841 (15)	0.64245 (14)	0.0280 (4)	
C2D	0.2393 (2)	0.82565 (16)	0.49213 (15)	0.0343 (4)	
C3D	0.2279 (17)	0.9296 (11)	0.3921 (11)	0.0446 (16)	0.499 (5)
F1D	0.2101 (9)	1.0139 (4)	0.4116 (5)	0.112 (3)	0.499 (5)
F2D	0.3266 (4)	0.9244 (3)	0.3131 (3)	0.0764 (15)	0.499 (5)
F3D	0.1208 (4)	0.9442 (4)	0.3547 (4)	0.087 (2)	0.499 (5)
C3D'	0.2036 (17)	0.9381 (11)	0.4023 (11)	0.0446 (16)	0.501 (5)
F1D'	0.2156 (10)	0.9282 (4)	0.3157 (3)	0.116 (3)	0.501 (5)
F2D'	0.0860 (4)	1.0024 (3)	0.4237 (4)	0.0802 (16)	0.501 (5)
F3D'	0.2875 (4)	0.9973 (4)	0.3863 (5)	0.0759 (18)	0.501 (5)
S1E	1.01005 (4)	0.44778 (4)	0.86856 (4)	0.03126 (12)	
O1E	0.94784 (14)	0.58947 (12)	0.67573 (11)	0.0405 (3)	
N1E	0.79039 (15)	0.50383 (13)	0.80787 (13)	0.0314 (3)	
H1E	0.7261 (19)	0.5320 (17)	0.7778 (17)	0.038*	
N2E	0.77016 (15)	0.42897 (13)	0.91058 (13)	0.0312 (3)	
C1E	0.91157 (18)	0.52690 (15)	0.76578 (15)	0.0293 (4)	
C2E	0.87636 (18)	0.39383 (14)	0.95031 (14)	0.0280 (4)	
C3E	0.8872 (13)	0.3062 (9)	1.0617 (6)	0.0361 (6)	0.69 (3)
F1E	0.9321 (9)	0.3380 (7)	1.1172 (7)	0.0522 (13)	0.69 (3)
F2E	0.9686 (10)	0.2101 (6)	1.0618 (7)	0.0684 (17)	0.69 (3)
F3E	0.7664 (7)	0.2929 (10)	1.1172 (8)	0.0677 (19)	0.69 (3)
C3E'	0.880 (3)	0.308 (2)	1.0615 (14)	0.0361 (6)	0.31 (3)
F1E'	0.926 (3)	0.2096 (11)	1.0533 (14)	0.072 (4)	0.31 (3)
F2E'	0.7658 (15)	0.3082 (18)	1.1266 (18)	0.051 (3)	0.31 (3)
F3E'	0.965 (2)	0.312 (2)	1.1065 (16)	0.063 (4)	0.31 (3)
S1F	0.58836 (5)	0.88473 (4)	0.44640 (4)	0.03321 (12)	
O1F	0.62033 (14)	0.68089 (11)	0.60648 (11)	0.0388 (3)	
N1F	0.79719 (17)	0.75904 (14)	0.52504 (13)	0.0367 (4)	
H1F	0.849 (2)	0.7086 (16)	0.5639 (17)	0.044*	
N2F	0.83663 (16)	0.85025 (14)	0.44911 (13)	0.0364 (4)	
C1F	0.66894 (18)	0.75638 (15)	0.54119 (14)	0.0296 (4)	
C2F	0.73824 (18)	0.92081 (15)	0.40304 (14)	0.0298 (4)	
C3F	0.7544 (3)	1.0293 (2)	0.3131 (2)	0.0362 (5)	0.908 (2)
F1F	0.7885 (3)	1.02172 (14)	0.21920 (11)	0.0763 (7)	0.908 (2)
F2F	0.64084 (16)	1.11017 (12)	0.31281 (15)	0.0615 (5)	0.908 (2)
F3F	0.84528 (19)	1.06373 (14)	0.32310 (15)	0.0662 (5)	0.908 (2)
C3F'	0.759 (2)	1.0237 (17)	0.3096 (18)	0.0362 (5)	0.092 (2)
F1F'	0.718 (3)	1.1043 (14)	0.3427 (12)	0.0763 (7)	0.092 (2)
F2F'	0.8847 (15)	1.0179 (13)	0.2641 (15)	0.0615 (5)	0.092 (2)
F3F'	0.6761 (19)	1.0648 (15)	0.2436 (15)	0.0662 (5)	0.092 (2)

### Atomic displacement parameters (Å^2^)

**Table:**

	*U*^11^	*U*^22^	*U*^33^	*U*^12^	*U*^13^	*U*^23^
S1A	0.0256 (2)	0.0354 (2)	0.0310 (2)	−0.01036 (19)	−0.00812 (18)	0.00015 (19)
O1A	0.0352 (7)	0.0419 (8)	0.0344 (7)	−0.0201 (6)	−0.0069 (6)	0.0029 (6)
N1A	0.0300 (8)	0.0348 (8)	0.0300 (8)	−0.0151 (7)	−0.0127 (7)	0.0062 (7)
N2A	0.0319 (8)	0.0372 (9)	0.0322 (8)	−0.0176 (7)	−0.0099 (7)	0.0031 (7)
C1A	0.0299 (9)	0.0288 (8)	0.0245 (8)	−0.0112 (7)	−0.0072 (7)	−0.0036 (7)
C2A	0.0290 (9)	0.0304 (9)	0.0245 (8)	−0.0118 (7)	−0.0056 (7)	−0.0033 (7)
C3A	0.0385 (12)	0.0386 (11)	0.0327 (11)	−0.0159 (10)	−0.0089 (9)	0.0040 (9)
F1A	0.176 (5)	0.108 (3)	0.0278 (12)	−0.085 (3)	−0.0336 (19)	0.0169 (14)
F2A	0.094 (3)	0.0308 (12)	0.099 (3)	−0.0039 (15)	0.022 (2)	0.0103 (13)
F3A	0.0565 (16)	0.088 (2)	0.080 (3)	−0.0527 (17)	−0.0287 (16)	0.0425 (19)
C3A'	0.0385 (12)	0.0386 (11)	0.0327 (11)	−0.0159 (10)	−0.0089 (9)	0.0040 (9)
F1A'	0.16 (1)	0.047 (4)	0.042 (3)	−0.057 (5)	−0.021 (5)	0.004 (3)
F2A'	0.064 (5)	0.046 (4)	0.061 (5)	−0.005 (4)	0.027 (4)	0.012 (3)
F3A'	0.064 (5)	0.060 (5)	0.058 (5)	−0.034 (4)	−0.039 (4)	0.027 (3)
S1B	0.0244 (2)	0.0297 (2)	0.0311 (2)	−0.01107 (18)	−0.01094 (18)	0.00303 (18)
O1B	0.0392 (8)	0.0442 (8)	0.0402 (8)	−0.0244 (7)	−0.0213 (6)	0.0140 (6)
N1B	0.0224 (7)	0.0317 (8)	0.0298 (8)	−0.0101 (6)	−0.0082 (6)	−0.0042 (6)
N2B	0.0282 (8)	0.0332 (8)	0.0295 (8)	−0.0137 (6)	−0.0034 (6)	−0.0064 (6)
C1B	0.0263 (9)	0.0291 (9)	0.0309 (9)	−0.0097 (7)	−0.0121 (7)	−0.0017 (7)
C2B	0.0290 (9)	0.0269 (8)	0.0277 (9)	−0.0118 (7)	−0.0036 (7)	−0.0048 (7)
C3B	0.0402 (13)	0.034 (1)	0.0306 (11)	−0.0157 (9)	−0.0072 (10)	−0.0006 (9)
F1B	0.085 (2)	0.0696 (17)	0.0473 (14)	−0.0416 (16)	−0.0370 (14)	0.0152 (11)
F2B	0.176 (4)	0.0276 (10)	0.0484 (13)	−0.0149 (16)	−0.024 (2)	−0.0022 (9)
F3B	0.0469 (12)	0.091 (2)	0.0511 (15)	−0.0233 (12)	0.0028 (10)	0.0301 (14)
C3B'	0.0402 (13)	0.034 (1)	0.0306 (11)	−0.0157 (9)	−0.0072 (10)	−0.0006 (9)
F1B'	0.075 (7)	0.056 (6)	0.063 (6)	−0.049 (5)	−0.037 (5)	0.032 (4)
F2B'	0.137 (11)	0.053 (5)	0.036 (4)	−0.018 (7)	−0.029 (6)	−0.013 (4)
F3B'	0.060 (5)	0.035 (5)	0.070 (6)	0.005 (3)	−0.017 (4)	0.014 (4)
S1C	0.0280 (2)	0.0413 (3)	0.0296 (2)	−0.0141 (2)	−0.01040 (18)	0.0006 (2)
O1C	0.0328 (7)	0.0437 (8)	0.0311 (7)	−0.0191 (6)	−0.0046 (6)	0.0013 (6)
N1C	0.0303 (8)	0.0344 (8)	0.0259 (8)	−0.0143 (7)	−0.0109 (6)	0.0024 (6)
N2C	0.0308 (8)	0.0344 (8)	0.0306 (8)	−0.0151 (7)	−0.0078 (7)	−0.0010 (7)
C1C	0.0263 (9)	0.0291 (9)	0.0264 (9)	−0.0092 (7)	−0.0057 (7)	−0.0054 (7)
C2C	0.0271 (9)	0.0294 (9)	0.0275 (9)	−0.0097 (7)	−0.0047 (7)	−0.0049 (7)
C3C	0.0377 (11)	0.0379 (11)	0.0316 (12)	−0.0139 (9)	−0.0066 (9)	−0.0003 (9)
F1C	0.112 (2)	0.0858 (19)	0.0287 (10)	−0.0521 (18)	−0.0219 (12)	0.0062 (10)
F2C	0.151 (4)	0.0261 (10)	0.0597 (17)	−0.0153 (14)	0.0255 (19)	−0.002 (1)
F3C	0.0472 (12)	0.113 (3)	0.0635 (18)	−0.0458 (14)	−0.0143 (11)	0.0371 (16)
C3C'	0.0377 (11)	0.0379 (11)	0.0316 (12)	−0.0139 (9)	−0.0066 (9)	−0.0003 (9)
F1C'	0.111 (8)	0.062 (5)	0.043 (4)	−0.066 (6)	−0.015 (5)	0.007 (3)
F2C'	0.114 (8)	0.039 (4)	0.042 (4)	−0.018 (5)	0.024 (5)	−0.008 (3)
F3C'	0.052 (4)	0.072 (7)	0.066 (6)	−0.017 (4)	−0.027 (4)	0.035 (5)
S1D	0.0316 (2)	0.0360 (3)	0.0367 (3)	−0.0148 (2)	−0.0166 (2)	0.0028 (2)
O1D	0.0348 (7)	0.0359 (7)	0.0326 (7)	−0.0158 (6)	−0.0154 (6)	0.0020 (6)
N1D	0.0236 (8)	0.0372 (8)	0.0312 (8)	−0.0114 (7)	−0.0083 (6)	−0.0059 (7)
N2D	0.0320 (8)	0.0407 (9)	0.0331 (9)	−0.0182 (7)	−0.0032 (7)	−0.0056 (7)
C1D	0.0258 (9)	0.0318 (9)	0.0243 (8)	−0.0091 (7)	−0.0073 (7)	−0.0061 (7)
C2D	0.0358 (10)	0.0347 (10)	0.0285 (9)	−0.0163 (8)	−0.0053 (8)	−0.0036 (8)
C3D	0.044 (5)	0.0404 (18)	0.037 (2)	−0.019 (2)	−0.009 (2)	0.0020 (16)
F1D	0.234 (9)	0.0336 (19)	0.068 (3)	−0.025 (4)	−0.056 (5)	−0.0092 (19)
F2D	0.064 (2)	0.069 (2)	0.0406 (18)	−0.0176 (18)	0.0047 (16)	0.0153 (15)
F3D	0.064 (2)	0.088 (3)	0.066 (3)	−0.038 (2)	−0.040 (2)	0.039 (2)
C3D'	0.044 (5)	0.0404 (18)	0.037 (2)	−0.019 (2)	−0.009 (2)	0.0020 (16)
F1D'	0.255 (9)	0.059 (2)	0.038 (2)	−0.043 (5)	−0.054 (4)	−0.0030 (18)
F2D'	0.060 (2)	0.0460 (19)	0.089 (3)	−0.0044 (15)	−0.0225 (19)	0.0116 (18)
F3D'	0.068 (2)	0.047 (3)	0.089 (4)	−0.035 (2)	−0.034 (2)	0.023 (2)
S1E	0.0248 (2)	0.0340 (2)	0.0298 (2)	−0.01272 (18)	−0.00930 (17)	−0.00089 (18)
O1E	0.0323 (7)	0.0462 (8)	0.0326 (7)	−0.0175 (6)	−0.0125 (6)	0.0039 (6)
N1E	0.0236 (8)	0.0353 (8)	0.0323 (8)	−0.0104 (6)	−0.0086 (6)	−0.0059 (7)
N2E	0.0262 (8)	0.0325 (8)	0.0331 (8)	−0.0121 (6)	−0.0028 (6)	−0.0095 (7)
C1E	0.0244 (8)	0.0299 (9)	0.0305 (9)	−0.0088 (7)	−0.0084 (7)	−0.0053 (7)
C2E	0.0268 (9)	0.0265 (8)	0.0299 (9)	−0.0113 (7)	−0.0033 (7)	−0.0080 (7)
C3E	0.0355 (16)	0.0319 (10)	0.0338 (10)	−0.0124 (10)	−0.0049 (9)	−0.0047 (8)
F1E	0.071 (3)	0.053 (2)	0.0369 (16)	−0.022 (2)	−0.020 (2)	−0.0093 (14)
F2E	0.089 (4)	0.0332 (16)	0.049 (2)	0.0112 (18)	−0.016 (2)	−0.0081 (13)
F3E	0.048 (2)	0.091 (5)	0.041 (3)	−0.043 (3)	−0.0090 (19)	0.014 (2)
C3E'	0.0355 (16)	0.0319 (10)	0.0338 (10)	−0.0124 (10)	−0.0049 (9)	−0.0047 (8)
F1E'	0.123 (10)	0.023 (3)	0.041 (4)	−0.013 (5)	−0.015 (6)	0.004 (3)
F2E'	0.048 (5)	0.051 (4)	0.033 (4)	−0.013 (4)	0.010 (4)	−0.010 (3)
F3E'	0.061 (6)	0.076 (9)	0.043 (5)	−0.038 (6)	−0.021 (5)	0.009 (4)
S1F	0.0259 (2)	0.0324 (2)	0.0338 (2)	−0.00987 (18)	−0.00592 (18)	−0.00442 (19)
O1F	0.0346 (7)	0.0387 (7)	0.0349 (7)	−0.0186 (6)	−0.0050 (6)	−0.0010 (6)
N1F	0.0307 (8)	0.0396 (9)	0.0290 (8)	−0.0158 (7)	−0.0110 (7)	0.0047 (7)
N2F	0.0332 (8)	0.0418 (9)	0.0283 (8)	−0.0187 (7)	−0.0079 (7)	0.0001 (7)
C1F	0.0286 (9)	0.0327 (9)	0.0247 (9)	−0.0110 (7)	−0.0046 (7)	−0.0065 (7)
C2F	0.0305 (9)	0.0348 (9)	0.0225 (8)	−0.0144 (8)	−0.0032 (7)	−0.0060 (7)
C3F	0.0387 (10)	0.0366 (10)	0.0297 (10)	−0.0167 (9)	−0.0051 (8)	−0.0052 (8)
F1F	0.141 (2)	0.0501 (9)	0.0212 (7)	−0.0369 (11)	0.0040 (9)	−0.0042 (6)
F2F	0.0500 (9)	0.0390 (8)	0.0646 (11)	−0.0093 (7)	−0.0085 (8)	0.0026 (7)
F3F	0.0679 (11)	0.0567 (10)	0.0703 (11)	−0.0422 (9)	−0.0275 (9)	0.0095 (8)
C3F'	0.0387 (10)	0.0366 (10)	0.0297 (10)	−0.0167 (9)	−0.0051 (8)	−0.0052 (8)
F1F'	0.141 (2)	0.0501 (9)	0.0212 (7)	−0.0369 (11)	0.0040 (9)	−0.0042 (6)
F2F'	0.0500 (9)	0.0390 (8)	0.0646 (11)	−0.0093 (7)	−0.0085 (8)	0.0026 (7)
F3F'	0.0679 (11)	0.0567 (10)	0.0703 (11)	−0.0422 (9)	−0.0275 (9)	0.0095 (8)

### Geometric parameters (Å, º)

**Table:**

S1A—C2A	1.7279 (18)	S1D—C2D	1.7262 (19)
S1A—C1A	1.7825 (18)	S1D—C1D	1.7728 (17)
O1A—C1A	1.213 (2)	O1D—C1D	1.214 (2)
N1A—C1A	1.355 (2)	N1D—C1D	1.355 (2)
N1A—N2A	1.358 (2)	N1D—N2D	1.357 (2)
N1A—H1A	0.819 (16)	N1D—H1D	0.821 (15)
N2A—C2A	1.277 (2)	N2D—C2D	1.279 (3)
C2A—C3A'	1.497 (13)	C2D—C3D	1.501 (9)
C2A—C3A	1.500 (5)	C2D—C3D'	1.516 (9)
C3A—F3A	1.289 (12)	C3D—F1D	1.26 (2)
C3A—F1A	1.295 (12)	C3D—F2D	1.284 (15)
C3A—F2A	1.296 (12)	C3D—F3D	1.34 (2)
C3A'—F3A'	1.29 (3)	C3D'—F1D'	1.25 (2)
C3A'—F2A'	1.29 (3)	C3D'—F2D'	1.293 (15)
C3A'—F1A'	1.29 (3)	C3D'—F3D'	1.35 (2)
S1B—C2B	1.7251 (18)	S1E—C2E	1.7262 (18)
S1B—C1B	1.7725 (17)	S1E—C1E	1.7764 (18)
O1B—C1B	1.213 (2)	O1E—C1E	1.214 (2)
N1B—N2B	1.357 (2)	N1E—C1E	1.357 (2)
N1B—C1B	1.358 (2)	N1E—N2E	1.360 (2)
N1B—H1B	0.826 (15)	N1E—H1E	0.814 (15)
N2B—C2B	1.282 (2)	N2E—C2E	1.281 (2)
C2B—C3B'	1.481 (15)	C2E—C3E'	1.490 (13)
C2B—C3B	1.509 (4)	C2E—C3E	1.506 (6)
C3B—F2B	1.294 (7)	C3E—F2E	1.301 (12)
C3B—F3B	1.305 (7)	C3E—F3E	1.329 (11)
C3B—F1B	1.317 (7)	C3E—F1E	1.333 (12)
C3B'—F2B'	1.27 (2)	C3E'—F2E'	1.29 (2)
C3B'—F3B'	1.28 (2)	C3E'—F3E'	1.31 (2)
C3B'—F1B'	1.29 (2)	C3E'—F1E'	1.32 (3)
S1C—C2C	1.7261 (18)	S1F—C2F	1.7297 (18)
S1C—C1C	1.7835 (18)	S1F—C1F	1.7881 (18)
O1C—C1C	1.211 (2)	O1F—C1F	1.213 (2)
N1C—N2C	1.357 (2)	N1F—C1F	1.354 (2)
N1C—C1C	1.358 (2)	N1F—N2F	1.357 (2)
N1C—H1C	0.829 (15)	N1F—H1F	0.820 (16)
N2C—C2C	1.279 (2)	N2F—C2F	1.275 (2)
C2C—C3C'	1.485 (14)	C2F—C3F'	1.484 (17)
C2C—C3C	1.506 (4)	C2F—C3F	1.505 (3)
C3C—F2C	1.283 (8)	C3F—F1F	1.300 (4)
C3C—F3C	1.299 (8)	C3F—F3F	1.319 (4)
C3C—F1C	1.317 (7)	C3F—F2F	1.326 (4)
C3C'—F2C'	1.27 (2)	C3F'—F1F'	1.28 (2)
C3C'—F3C'	1.28 (2)	C3F'—F3F'	1.30 (2)
C3C'—F1C'	1.29 (2)	C3F'—F2F'	1.30 (2)
			
C2A—S1A—C1A	88.14 (8)	C2D—S1D—C1D	87.99 (9)
C1A—N1A—N2A	119.32 (15)	C1D—N1D—N2D	118.36 (15)
C1A—N1A—H1A	119.3 (16)	C1D—N1D—H1D	122.7 (16)
N2A—N1A—H1A	121.2 (16)	N2D—N1D—H1D	118.9 (16)
C2A—N2A—N1A	109.19 (15)	C2D—N2D—N1D	109.65 (15)
O1A—C1A—N1A	126.00 (17)	O1D—C1D—N1D	127.28 (16)
O1A—C1A—S1A	127.78 (15)	O1D—C1D—S1D	125.59 (14)
N1A—C1A—S1A	106.22 (12)	N1D—C1D—S1D	107.14 (13)
N2A—C2A—C3A'	121.6 (12)	N2D—C2D—C3D	117.0 (8)
N2A—C2A—C3A	120.8 (5)	N2D—C2D—C3D'	123.4 (8)
N2A—C2A—S1A	117.06 (14)	N2D—C2D—S1D	116.86 (14)
C3A'—C2A—S1A	121.2 (12)	C3D—C2D—S1D	125.9 (9)
C3A—C2A—S1A	122.1 (5)	C3D'—C2D—S1D	119.7 (8)
F3A—C3A—F1A	107.9 (8)	F1D—C3D—F2D	110.1 (14)
F3A—C3A—F2A	109.2 (8)	F1D—C3D—F3D	108.4 (10)
F1A—C3A—F2A	107.7 (8)	F2D—C3D—F3D	103.9 (14)
F3A—C3A—C2A	112.0 (7)	F1D—C3D—C2D	111.5 (13)
F1A—C3A—C2A	110.0 (7)	F2D—C3D—C2D	114.5 (9)
F2A—C3A—C2A	109.9 (7)	F3D—C3D—C2D	107.9 (12)
F3A'—C3A'—F2A'	104.3 (17)	F1D'—C3D'—F2D'	109.6 (14)
F3A'—C3A'—F1A'	103.3 (17)	F1D'—C3D'—F3D'	108.4 (10)
F2A'—C3A'—F1A'	104.3 (18)	F2D'—C3D'—F3D'	102.7 (14)
F3A'—C3A'—C2A	115.2 (19)	F1D'—C3D'—C2D	111.5 (13)
F2A'—C3A'—C2A	114.1 (18)	F2D'—C3D'—C2D	114.9 (9)
F1A'—C3A'—C2A	114.4 (18)	F3D'—C3D'—C2D	109.0 (13)
C2B—S1B—C1B	87.86 (8)	C2E—S1E—C1E	88.10 (8)
N2B—N1B—C1B	118.20 (15)	C1E—N1E—N2E	118.65 (15)
N2B—N1B—H1B	119.2 (15)	C1E—N1E—H1E	126.0 (16)
C1B—N1B—H1B	122.4 (15)	N2E—N1E—H1E	115.3 (16)
C2B—N2B—N1B	109.50 (15)	C2E—N2E—N1E	109.35 (15)
O1B—C1B—N1B	127.14 (16)	O1E—C1E—N1E	127.58 (17)
O1B—C1B—S1B	125.52 (14)	O1E—C1E—S1E	125.56 (14)
N1B—C1B—S1B	107.33 (12)	N1E—C1E—S1E	106.86 (13)
N2B—C2B—C3B'	122.3 (10)	N2E—C2E—C3E'	119.3 (11)
N2B—C2B—C3B	120.5 (3)	N2E—C2E—C3E	121.9 (5)
N2B—C2B—S1B	117.11 (14)	N2E—C2E—S1E	117.02 (14)
C3B'—C2B—S1B	120.5 (10)	C3E'—C2E—S1E	123.7 (11)
C3B—C2B—S1B	122.4 (3)	C3E—C2E—S1E	121.0 (5)
F2B—C3B—F3B	108.9 (5)	F2E—C3E—F3E	109.1 (9)
F2B—C3B—F1B	106.6 (5)	F2E—C3E—F1E	107.0 (8)
F3B—C3B—F1B	106.2 (5)	F3E—C3E—F1E	105.9 (9)
F2B—C3B—C2B	111.7 (4)	F2E—C3E—C2E	113.5 (9)
F3B—C3B—C2B	112.1 (4)	F3E—C3E—C2E	110.5 (9)
F1B—C3B—C2B	111.0 (4)	F1E—C3E—C2E	110.5 (8)
F2B'—C3B'—F3B'	106.6 (18)	F2E'—C3E'—F3E'	109 (2)
F2B'—C3B'—F1B'	109.8 (18)	F2E'—C3E'—F1E'	106 (2)
F3B'—C3B'—F1B'	105.5 (16)	F3E'—C3E'—F1E'	106.1 (19)
F2B'—C3B'—C2B	110.7 (16)	F2E'—C3E'—C2E	116 (2)
F3B'—C3B'—C2B	112.6 (16)	F3E'—C3E'—C2E	111.9 (19)
F1B'—C3B'—C2B	111.4 (15)	F1E'—C3E'—C2E	107.3 (18)
C2C—S1C—C1C	88.23 (8)	C2F—S1F—C1F	88.00 (9)
N2C—N1C—C1C	119.38 (15)	C1F—N1F—N2F	119.42 (16)
N2C—N1C—H1C	118.0 (16)	C1F—N1F—H1F	120.7 (17)
C1C—N1C—H1C	122.4 (16)	N2F—N1F—H1F	119.7 (17)
C2C—N2C—N1C	109.16 (15)	C2F—N2F—N1F	109.29 (15)
O1C—C1C—N1C	125.55 (17)	O1F—C1F—N1F	126.26 (17)
O1C—C1C—S1C	128.35 (14)	O1F—C1F—S1F	127.58 (15)
N1C—C1C—S1C	106.10 (12)	N1F—C1F—S1F	106.16 (13)
N2C—C2C—C3C'	118.9 (10)	N2F—C2F—C3F'	120 (1)
N2C—C2C—C3C	120.8 (3)	N2F—C2F—C3F	120.8 (2)
N2C—C2C—S1C	117.10 (14)	N2F—C2F—S1F	117.12 (14)
C3C'—C2C—S1C	123.7 (10)	C3F'—C2F—S1F	122.7 (10)
C3C—C2C—S1C	122.0 (3)	C3F—C2F—S1F	122.07 (17)
F2C—C3C—F3C	111.2 (5)	F1F—C3F—F3F	108.5 (3)
F2C—C3C—F1C	106.7 (5)	F1F—C3F—F2F	107.2 (3)
F3C—C3C—F1C	105.0 (5)	F3F—C3F—F2F	105.7 (3)
F2C—C3C—C2C	110.8 (5)	F1F—C3F—C2F	111.7 (2)
F3C—C3C—C2C	111.8 (5)	F3F—C3F—C2F	112.0 (2)
F1C—C3C—C2C	111.0 (4)	F2F—C3F—C2F	111.4 (2)
F2C'—C3C'—F3C'	101.9 (16)	F1F'—C3F'—F3F'	97.7 (18)
F2C'—C3C'—F1C'	103.9 (17)	F1F'—C3F'—F2F'	106 (2)
F3C'—C3C'—F1C'	107.0 (17)	F3F'—C3F'—F2F'	115.2 (19)
F2C'—C3C'—C2C	113.3 (17)	F1F'—C3F'—C2F	108.7 (18)
F3C'—C3C'—C2C	113.8 (17)	F3F'—C3F'—C2F	114.3 (17)
F1C'—C3C'—C2C	115.6 (16)	F2F'—C3F'—C2F	113.6 (16)
			
C1A—N1A—N2A—C2A	1.5 (3)	C1D—N1D—N2D—C2D	0.5 (3)
N2A—N1A—C1A—O1A	177.87 (19)	N2D—N1D—C1D—O1D	178.44 (19)
N2A—N1A—C1A—S1A	−2.7 (2)	N2D—N1D—C1D—S1D	−1.0 (2)
C2A—S1A—C1A—O1A	−178.3 (2)	C2D—S1D—C1D—O1D	−178.59 (19)
C2A—S1A—C1A—N1A	2.28 (14)	C2D—S1D—C1D—N1D	0.85 (14)
N1A—N2A—C2A—C3A'	−176.0 (14)	N1D—N2D—C2D—C3D	−174.6 (9)
N1A—N2A—C2A—C3A	−178.6 (6)	N1D—N2D—C2D—C3D'	176.2 (9)
N1A—N2A—C2A—S1A	0.6 (2)	N1D—N2D—C2D—S1D	0.2 (2)
C1A—S1A—C2A—N2A	−1.78 (17)	C1D—S1D—C2D—N2D	−0.65 (18)
C1A—S1A—C2A—C3A'	174.9 (14)	C1D—S1D—C2D—C3D	173.7 (9)
C1A—S1A—C2A—C3A	177.4 (6)	C1D—S1D—C2D—C3D'	−176.8 (9)
N2A—C2A—C3A—F3A	0.0 (11)	N2D—C2D—C3D—F1D	−73.1 (13)
S1A—C2A—C3A—F3A	−179.1 (6)	S1D—C2D—C3D—F1D	112.6 (14)
N2A—C2A—C3A—F1A	−120.0 (7)	N2D—C2D—C3D—F2D	53 (2)
S1A—C2A—C3A—F1A	60.9 (10)	S1D—C2D—C3D—F2D	−121.5 (14)
N2A—C2A—C3A—F2A	121.6 (7)	N2D—C2D—C3D—F3D	167.9 (8)
S1A—C2A—C3A—F2A	−57.5 (10)	S1D—C2D—C3D—F3D	−6.4 (16)
N2A—C2A—C3A'—F3A'	−167.5 (15)	N2D—C2D—C3D'—F1D'	106.3 (15)
S1A—C2A—C3A'—F3A'	16 (3)	S1D—C2D—C3D'—F1D'	−77.8 (13)
N2A—C2A—C3A'—F2A'	−47 (3)	N2D—C2D—C3D'—F2D'	−128.1 (14)
S1A—C2A—C3A'—F2A'	136.6 (18)	S1D—C2D—C3D'—F2D'	48 (2)
N2A—C2A—C3A'—F1A'	73 (2)	N2D—C2D—C3D'—F3D'	−13.4 (16)
S1A—C2A—C3A'—F1A'	−103 (2)	S1D—C2D—C3D'—F3D'	162.4 (7)
C1B—N1B—N2B—C2B	0.5 (2)	C1E—N1E—N2E—C2E	0.9 (2)
N2B—N1B—C1B—O1B	178.8 (2)	N2E—N1E—C1E—O1E	178.64 (19)
N2B—N1B—C1B—S1B	−0.7 (2)	N2E—N1E—C1E—S1E	−1.6 (2)
C2B—S1B—C1B—O1B	−179.0 (2)	C2E—S1E—C1E—O1E	−178.87 (19)
C2B—S1B—C1B—N1B	0.47 (14)	C2E—S1E—C1E—N1E	1.36 (14)
N1B—N2B—C2B—C3B'	175.6 (12)	N1E—N2E—C2E—C3E'	−178.1 (13)
N1B—N2B—C2B—C3B	179.9 (3)	N1E—N2E—C2E—C3E	−177.4 (6)
N1B—N2B—C2B—S1B	−0.1 (2)	N1E—N2E—C2E—S1E	0.3 (2)
C1B—S1B—C2B—N2B	−0.25 (16)	C1E—S1E—C2E—N2E	−1.04 (16)
C1B—S1B—C2B—C3B'	−176.0 (11)	C1E—S1E—C2E—C3E'	177.3 (14)
C1B—S1B—C2B—C3B	179.8 (3)	C1E—S1E—C2E—C3E	176.7 (6)
N2B—C2B—C3B—F2B	−92.6 (5)	N2E—C2E—C3E—F2E	108.4 (9)
S1B—C2B—C3B—F2B	87.3 (6)	S1E—C2E—C3E—F2E	−69.2 (11)
N2B—C2B—C3B—F3B	29.9 (6)	N2E—C2E—C3E—F3E	−14.5 (13)
S1B—C2B—C3B—F3B	−150.1 (4)	S1E—C2E—C3E—F3E	167.9 (8)
N2B—C2B—C3B—F1B	148.5 (4)	N2E—C2E—C3E—F1E	−131.3 (8)
S1B—C2B—C3B—F1B	−31.5 (6)	S1E—C2E—C3E—F1E	51.0 (12)
N2B—C2B—C3B'—F2B'	105.9 (18)	N2E—C2E—C3E'—F2E'	−29 (3)
S1B—C2B—C3B'—F2B'	−79 (2)	S1E—C2E—C3E'—F2E'	152.8 (17)
N2B—C2B—C3B'—F3B'	−134.9 (15)	N2E—C2E—C3E'—F3E'	−155 (2)
S1B—C2B—C3B'—F3B'	41 (2)	S1E—C2E—C3E'—F3E'	27 (3)
N2B—C2B—C3B'—F1B'	−17 (2)	N2E—C2E—C3E'—F1E'	89 (2)
S1B—C2B—C3B'—F1B'	158.9 (14)	S1E—C2E—C3E'—F1E'	−89 (2)
C1C—N1C—N2C—C2C	0.7 (3)	C1F—N1F—N2F—C2F	−0.9 (3)
N2C—N1C—C1C—O1C	179.06 (19)	N2F—N1F—C1F—O1F	−179.47 (19)
N2C—N1C—C1C—S1C	−1.6 (2)	N2F—N1F—C1F—S1F	1.2 (2)
C2C—S1C—C1C—O1C	−179.2 (2)	C2F—S1F—C1F—O1F	179.82 (19)
C2C—S1C—C1C—N1C	1.48 (14)	C2F—S1F—C1F—N1F	−0.88 (14)
N1C—N2C—C2C—C3C'	−173.3 (12)	N1F—N2F—C2F—C3F'	−174.3 (13)
N1C—N2C—C2C—C3C	−177.6 (4)	N1F—N2F—C2F—C3F	−178.5 (2)
N1C—N2C—C2C—S1C	0.6 (2)	N1F—N2F—C2F—S1F	0.1 (2)
C1C—S1C—C2C—N2C	−1.29 (17)	C1F—S1F—C2F—N2F	0.50 (17)
C1C—S1C—C2C—C3C'	172.3 (12)	C1F—S1F—C2F—C3F'	174.7 (13)
C1C—S1C—C2C—C3C	176.9 (4)	C1F—S1F—C2F—C3F	179.1 (2)
N2C—C2C—C3C—F2C	105.1 (6)	N2F—C2F—C3F—F1F	92.2 (3)
S1C—C2C—C3C—F2C	−73.0 (6)	S1F—C2F—C3F—F1F	−86.3 (3)
N2C—C2C—C3C—F3C	−19.6 (7)	N2F—C2F—C3F—F3F	−29.7 (4)
S1C—C2C—C3C—F3C	162.2 (4)	S1F—C2F—C3F—F3F	151.8 (2)
N2C—C2C—C3C—F1C	−136.5 (4)	N2F—C2F—C3F—F2F	−147.8 (2)
S1C—C2C—C3C—F1C	45.4 (7)	S1F—C2F—C3F—F2F	33.6 (3)
N2C—C2C—C3C'—F2C'	−82 (2)	N2F—C2F—C3F'—F1F'	−98.9 (19)
S1C—C2C—C3C'—F2C'	104.1 (19)	S1F—C2F—C3F'—F1F'	87 (2)
N2C—C2C—C3C'—F3C'	161.8 (15)	N2F—C2F—C3F'—F3F'	153.2 (17)
S1C—C2C—C3C'—F3C'	−12 (3)	S1F—C2F—C3F'—F3F'	−21 (3)
N2C—C2C—C3C'—F1C'	37 (2)	N2F—C2F—C3F'—F2F'	18 (3)
S1C—C2C—C3C'—F1C'	−136.1 (15)	S1F—C2F—C3F'—F2F'	−155.7 (15)

### Hydrogen-bond geometry (Å, º)

**Table:**

*D*—H···*A*	*D*—H	H···*A*	*D*···*A*	*D*—H···*A*
N1*A*—H1*A*···O1*B*	0.82 (2)	1.92 (2)	2.726 (2)	169 (2)
N1*B*—H1*B*···O1*A*	0.83 (2)	2.57 (2)	3.328 (2)	152 (2)
N1*B*—H1*B*···O1*A*^i^	0.83 (2)	2.35 (2)	2.955 (2)	131 (2)
N1*C*—H1*C*···O1*D*	0.83 (2)	1.93 (2)	2.7485 (19)	171 (2)
N1*D*—H1*D*···O1*C*	0.82 (2)	2.61 (2)	3.342 (2)	150 (2)
N1*D*—H1*D*···O1*F*	0.82 (2)	2.28 (2)	2.908 (2)	134 (2)
N1*E*—H1*E*···O1*C*	0.81 (2)	2.26 (2)	2.912 (2)	137 (2)
N1*E*—H1*E*···O1*F*	0.81 (2)	2.64 (2)	3.359 (2)	148 (2)
N1*F*—H1*F*···O1*E*	0.82 (2)	1.95 (2)	2.764 (2)	171 (2)

Symmetry code: (i) −*x*+2, −*y*+1, −*z*+1.
